# A lifetime of stress: ATF6 in development and homeostasis

**DOI:** 10.1186/s12929-018-0453-1

**Published:** 2018-05-25

**Authors:** Robert F. Hillary, Una FitzGerald

**Affiliations:** Galway Neuroscience Centre, Cúram Centre for Research in Medical Devices, School of Natural Sciences, National University of Ireland, Galway, Ireland

**Keywords:** Endoplasmic reticulum stress, Unfolded protein response, ATF6, Development, Homeostasis, Apoptosis

## Abstract

**Background:**

Activating transcription factor 6 (ATF6) is an endoplasmic reticulum (ER)-localised protein and member of the leucine zipper family of transcription factors. Best known for its role in transducing signals linked to stress to the endoplasmic reticulum, the 50 kDa activated form of ATF6 is now emerging as a major regulator of organogenesis and tissue homeostasis. Responsible for the correct folding, secretion and membrane insertion of a third of the proteome in eukaryotic cells, the ER encompasses a dynamic, labyrinthine network of regulators, chaperones, foldases and cofactors. Such structures are crucial to the extensive protein synthesis required to undergo normal development and maintenance of tissue homeostasis. When an additional protein synthesis burden is placed on the ER, ATF6, in tandem with ER stress transducers inositol requiring enzyme 1 (IRE1) and PKR-like endoplasmic reticulum kinase (PERK), slows the pace of protein translation and induces the production of stress-reducing chaperones and foldases.

**Main Text:**

In the context of development and tissue homeostasis, however, distinct cellular impacts have been attributed to ATF6. Drawing on data published from human, rodent, fish, goat and bovine research, this review first focuses on ATF6-mediated regulation of osteo- and chondrogenesis, ocular development as well as neuro- and myelinogenesis. The purported role of ATF6 in development of the muscular and reproductive systems as well as adipo- and lipogenesis is then described. With relevance to cardiac disease, cancer and brain disorders, the importance of ATF6 in maintaining tissue homeostasis is the subject of the final section.

**Conclusion:**

In conclusion, the review encourages further elucidation of ATF6 regulatory operations during organogenesis and tissue homeostasis, to spawn the development of ATF6-targeted therapeutic strategies.

## Background

Throughout their lifespan, cells endure a barrage of environmental stressors, such as hypoxia and pathogens, which may provoke cellular damage or even death. Consequently, cells retaliate by effectuating diverse evolutionarily conserved defence strategies in order to overcome the presented exogenous challenge. In recent years, endogenous reservoirs of cellular stress have attracted an accumulating wealth of interest and scrutiny [1]. A cardinal source of such stress rests in the endoplasmic reticulum (ER), a fundamental subcellular compartment which conducts disparate biochemical and metabolic operations (Table 1). This tubular network contributes to lipid biosynthesis, calcium homeostasis and the biogenesis of autophagosomes and peroxisomes. Moreover, the ER is responsible for the folding and maturation of one-third of the proteome in most eukaryotic cells, most of which are dedicated to the secretory pathway or membrane insertion [2]. Accordingly, the maintenance of ER protein homeostasis (proteostasis) encompasses a dynamic, labyrinthine network of regulators, chaperones, foldases and cofactors. The rapid progression of cell lineages during development requires extensive protein manufacture which must occur with high degrees of fidelity and efficiency. Indeed, in developmental contexts, the molecular machinery associated with the ER and protein folding is up-regulated and the ER itself undergoes expansion in order to prevent the accumulation of misfolded proteins. Developmental processes incite an overwhelming production of proteins (and lipids) and thus create environments conducive to ER stress as well as the induction of the associated cellular response pathway – the unfolded protein response (UPR) [3].

**Table 1:** Endoplasmic reticulum stress and the unfolded protein response

Under unstressed conditions, the chief regulators of the tripartite UPR network, PERK, IRE1 and ATF6, interact with binding immunoglobulin protein (BiP; also known as GRP78 and HSP5A) through their luminal domains, which stabilises these transmembrane proteins in an inactive state. Subsequent to cellular insults such as perturbed calcium homeostasis, the ER folding capacity may become overwhelmed, prompting an accumulation of aberrant protein species in the ER lumen. The malfolded secretory cargo sequesters BiP thereby liberating activation of the UPR cascade and encouraging efforts towards proteostasis restoration [58]. PERK and IRE1 are type I transmembrane proteins with cytosolic serine/threonine kinase domains which perform kinase (PERK and IRE1) and endoribonuclease (IRE1) activities during ER stress relief endeavours. Emancipation from BiP releases PERK and IRE1 for homo-oligomerisation and *trans*-phosphorylation eliciting their activation. Activated PERK targets and phosphorylates eukaryotic initiation factor 2α (eIF2α) at serine-51, which diminishes the presence of eIF2-guanosine triphosphate-tRNA^methionine^ ternary complexes, precipitating attenuation of global translation [59]. On the other hand, IRE1 splices a 26 base pair intron from *X-box binding protein 1 (Xbp1)* mRNA, generating a transcriptionally active XBP1 protein that regulates genes involved in protein trafficking, folding and cell survival [60]. ER stress also induces disunion of BiP from ATF6 (type II transmembrane glycoprotein), resulting in the exposure of Golgi-localisation sequences on the ER-luminal domain of ATF6. Following translocation to the Golgi apparatus, ATF6 is cleaved sequentially by site-1-protease and site-2-protease, liberating a 50 kDa amino-terminal cytoplasmic fragment (ATF6f). ATF6f enters the nucleus and binds to ER stress-response elements which results in the expression of ER stress proteins including BiP and XBP1 (thereby augmenting the output of the IRE1 arm). The activation of the PERK and ATF6 axes is thought to precede IRE1 activation, consistent with the proposed signals transduced by these branches. Indeed, the PERK and ATF6 pathways primarily are implicated in adaptive responses to protein misfolding whereas the IRE1 arm is associated with a complex dichotomy of promoting both survival and pro-apoptotic programmes [61].	

Cells respond to ER stress through activation and employment of the evolutionarily conserved, unfolded protein response. Mechanistically, the adaptive UPR signalling pathway attempts to attenuate proteostatic collapse through increasing chaperone expression, halting protein translation and degrading abnormally folded proteins. The UPR is commanded by three master regulators: protein kinase RNA-like endoplasmic reticulum kinase (PERK), inositol-requiring enzyme 1 (IRE1) and activating transcription factor 6 (ATF6). The PERK, IRE1 and ATF6 axes coordinate downstream components which initially promote cellular protective events; however, in the presence of chronic and unresolved stress, apoptotic mechanisms prevail to eliminate the stressed cells [4]. Recently, the UPR field has enjoyed exponential growth owing to multitudinous mechanistic in vitro studies attempting to define the molecular orchestra which frames restorative programmes following perturbations in proteostasis. However, the realisation that the UPR engages in complex crosstalk networks with distinct biochemical pathways has expanded the physiological repertoire of the UPR beyond abating aberrations in the protein load. Indeed, recent evidence has suggested that ER stress response mediators also participate in regulating other processes such as cellular differentiation and maturation. Furthermore, dysregulation of the UPR has been implicated in diverse pathological states including cancer, neurodegeneration and immune disorders [5]. Together, these novel insights have motivated enterprises which aim to elucidate the role of the ATF6 pathway in organogenesis and tissue homeostasis (Tables 2 and 3, respectively). Relative to other UPR arms, the significance of ATF6 in such processes has been neglected, despite exciting advancements [6]. Hence, these findings demand framework and rationalisation in order to expedite the elucidation of ATF6-related physiology and, potentially, to foster the development of ATF6-targeted therapeutic strategies. Therefore, here, we highlight and clarify current data pertinent to the role of ATF6 in development and tissue homeostasis, and the pathological consequences of ATF6 deregulation.

**Table 2:** The role of ATF6 in the development of diverse tissues and organs

Tissue	Intervention/Observation	Effect/Comment	Reference(s)
Bone and Cartilage	OE of ATF6 in foetal mouse metatarsals ex vivo as well as C3H10T1/2 and ATDC5 cells in vitro	Enhanced chondrocyte hypertrophy, mineralisation, and endochondral bone growth in metatarsals and enhanced chondrocyte differentiation in vitro	[17–19]
	KD of ATF6 in C3H10T1/2 and ATDC5 cells (adenoviral siRNA delivery system)	KD➔Inhibition of chondrocyte differentiation and hypertrophy	
	KD of ATF6α in murine bone marrow stromal cells and C3H10T1/2 cells	BMP2➔RunX2➔ATF6➔osteocalcin	
	MC3T3-E1 + MTA ATF6 KD in MTA-treated cells	MTA stimulated osteoblastic differentiation via upregulation of osteocalcin ATF6 KD abrogated MTA-induced mineralisation	[20]
	OE of ATF6 in human dental pulp cells	Matrix mineralisation and odontoblastic differentiation increased	[21]
Ocular Tissue	Immunofluorescent staining of ATF6 expression in the embryonic eye lens (FVB/N-Har mice)	ATF6 expression and cleavage were detected in lens fibre cells in the developing mouse embryo	[24]
	Homozygosity mapping, linkage analyses and exome sequencing in patients with achromatopsia (ACHM)	Patients with ACHM displayed *ATF6* mutations, which severely compromise ATF6 function. Patients also exhibited foveal deficits, suggesting that ATF6 plays a crucial role in the development of the fovea and cone photoreceptors in humans	[25–27]
	Early-onset photoreceptor degeneration	ATF6 splice-variant mutations causing compromised photoreceptor function	
Nervous tissue	Western blot analyses of glycosylated ATF6 expression in adult and embryonic mouse brains	Compared to adult tissue, the expression of partially glycosylated ATF6 is elevated in the brains of mouse embryos	[30]
	Olfactory sensory neurons	Low-level expression (relative to ATF5) of ATF6 during development	[32]
	Immunohistochemical staining of ATF6 (cytoplasmic and nuclear) in the developing rat cerebellum	ATF6 was activated at postnatal day 7 (prior to the appearance of myelin), with maximal nuclear-localised ATF6 visualised at postnatal day 10 (onset of myelination)	[33]
Muscle	OE of active ATF6 in myoblasts	↑ ATF6 ➔ ↓ Mcl-1 ➔ apoptotic myoblast cells. ATF6 may regulate muscle cell development through promoting post-translational modification of Mcl-1	[35]
	OE of DKK3 in mESCs Transient ATF6 overexpression or siRNA-mediated ATF6 KD at day 2 of ESC differentiation	DKK3 induced differentiation of ESCs into smooth muscle cells (SMCs) ↑ ATF6 during DKK3-induced ESC-SMC differentiation OE and KD ➔ ATF6 is involved in DKK3-mediated SMC generation via ↑myocardin expression	[36]
Ovarian Tissue	Analyses of ATF6 and associated markers during the bovine corpus luteum lifespan and in goat granulosa	ATF6 and ATF4➔ ↑CHOP and ↑caspase-12	[37–39]
	Molecular and immunohistochemical detection of ATF6α during peri-implantation and the oestrous cycle in mice	↑ATF6α mRNA and protein in the d5 uterus close to the implantation site and in d7–8 secondary decidual zone; ATF6α expression affected by progesterone and estrogen in ovariectomised mice	[40]
Adipose Tissue	KD of ATF6α in C3H10T1/2 cells	ATF6α KD ➔ ↓ C3H10T1/2 differentiation and ↓ lipid accumulation	[41]
	Glucose deprivation	↓SREBP2-mediated lipogenesis	[42]
	Adipogenesis in salmon and rainbow trout	ATF6/ATF6β upregulated during adipogenesis	[14, 43]
	OE and KD in pre-adipocytic 3 T3-L1 cells	ATF6 OE ➔ ↑TIS7 in 3T3-L1 cells ATF6KD under hypoxia➔↑AP-2 in pre-adipocytes; ATF6 KD ↓ hypoxia-induced ↑TIS7 and ↓adipogenic gene expression	[44]
Early Stem Cell/Mesoderm	Small molecule ATF6 activation in human ESCs	ATF6 activation suppressed pluripotency, enhanced stem cell differentiation and steered cells towards mesodermal fate	[28]

*Key*: *ACHM* achromatopsia, *ATF6* activating transcription factor 6, *CHOP* C/EBP homologous protein, *ESC* embryonic stem cell, *KD* knockdown, *KO* knockout, *MCL-1* myeloid cell leukaemia sequence 1, *MTA* mineral trioxide aggregate, *OE* over-expression, *PCR* polymerase chain reaction, *RunX2* runt-related transcription factor 2, *SMC* smooth muscle cell, *SREBP-2* sterol regulatory element binding protein 2, *TIS7* tetradecanoyl phorbol acetate induced sequence 7

**Table 3:** ATF6 signalling in tissue homeostasis and pathogenesis

Tissue	Model	Effect	Reference
Protective Role of ATF6 Signalling			
Heart	Mouse model of myocardial ischemia/reperfusion damage KO of ATF6 in mice	When compared to wild-type tissue, ATF6 KO mouse cardiac tissue exhibited increased damage upon ischemia/reperfusion. Mechanistically, ATF6 upregulates oxidative stress genes, such as catalase, to exert cardioprotective effects in this context	[49]
Kidney	Tunicamycin-induced cytotoxicity in rat-derived glomerular epithelial cells (GECs)	Calcium-independent phospholipase A2γ (iPLA2γ) is protective against GEC injury. ATF6 contributes to iPLA2γ-mediated cytoprotection	[50]
Brain	Short-form ATF6 KI in forebrain neurons of mice (tamoxifen-inducible activation) Experimental model of stroke induced by middle cerebral artery occlusion	Forced activation of ATF6 reduced infarct volume and improved functional outcome following 24 h post-model induction	[51]
	R6/2 mouse model of Huntington’s disease	Derepression of ATF6 was associated with early neuroprotection in this model of Huntington’s disease	[52]
	Kainate-induced neurotoxicity in hippocampi of mice ATF6α KO	Kainate induced pronounced neuronal death in hippocampal CA3 region of ATF6α-KO mice. Hence, ATF6α protects against kainate-induced neurotoxicity in mice	[53]
Pancreas/Liver	Diet-induced obese mice KOt of ATF6α	ATF6α protects pancreatic β-cells from endoplasmic reticulum stress	[55]
	Zebrafish model of endoplasmic reticulum stress and fatty liver disease Depletion of active ATF6 through mutation in site-1 protease gene (*mbtps1*) or *atf6* morpholino injection	ATF6 protects against hepatic steatosis following tunicamycin-induced acute endoplasmic reticulum stress	[57]
Pathological Role of ATF6 Signalling			
Liver	OE of activated form of ATF6α in human hepatocellular carcinoma cell line (HLF)	ATF6α maylead to hepatocarcinogenesis by directly and indirectly regulating a broad range of genes associated with transformation	[46]
	Zebrafish model of endoplasmic reticulum stress and fatty liver disease; Depletion of active ATF6 through mutation in site-1 protease gene (*mbtps1*) or *atf6* morpholino injection	ATF6 ➔ hepatic steatosis resulting from chronic endoplasmic reticulum stress	[57]
Squamous Epithelium	Quiescent human squamous carcinoma cells (D-HEp3 cells) KD of ATF6α expression in D-Hep3 cells using chick chorioallantoic membrane and nude mice for xenograft studies	ATF6α ➔ dormant cell survival, adaptation of dormant cells to chemotherapy, nutritional stress and the in vivo microenvironment ATF6α-Rheb-mTOR signalling ➔ survival and adaptation of carcinoma cells	[47]
Pancreas	Diet-induced obese mice KO of ATF6α	ATF6α ➔ development of hyperlipidaemia and insulin resistance in mouse model of diabetes	[55]
	Otsuka Long Evans Tokushima Fatty rat model of type II diabetes	↑ ATF6 in pancreatic islets in diseased rats; ATF6 ➔ ↓insulin levels	[56]

*Key: ATF6* activating transcription factor 6, *GEC* glomerular epithelial cell, *iPLA2γ* calcium-independent phospholipase A2γ, *KD* knockdown, *KI* knockin, *KO* knockout

### The ATF6 axis of the unfolded protein response

Mammals express two homologous ATF6 proteins, ATF6α (670 amino acids) and ATF6β (703 amino acids); the biochemical and physiological characteristics of the former are significantly better documented than the latter. The C-termini of ATF6 isoforms protrude into the ER lumen, whereas the N-termini face the cytosol. The cytoplasmic portion of ATF6 encompasses basic leucine zipper (bZIP) DNA binding and transcriptional activation domains, which are followed by a 20-amino acid transmembrane domain. Interestingly, although ATF6α and ATF6β possess significant sequence homology, these isoforms exhibit divergent transcriptional activation domains. Indeed, ATF6α is a potent transcriptional activator whereas ATF6β, a poor transcriptional activator, may inhibit activation by ATF6α [7]. In contrast with the protein kinases PERK and IRE1 (type I transmembrane proteins), ATF6α is a 90 kDa type II transmembrane glycoprotein and member of the bZIP transcription factor family [8]. The ER stress-induced disunion of binding immunoglobulin protein (BiP) from ATF6α exposes two Golgi-localisation sequences within the ER-luminal domain of ATF6α (GLS1 and GLS2 corresponding to residues 468–475 and 476–500, respectively), evoking its translocation to the Golgi apparatus and cleavage by two proteases therein [9]. Site-1 protease (S1P) and S2P sequentially remove the luminal domain and the transmembrane anchor, respectively, mobilising a 50 kDa amino-terminal cytoplasmic fragment (ATF6f). Following such regulated intramembrane proteolysis (RIP), the liberated ATF6f transcription factor enters the nucleus and binds to ER stress-response elements [4]. Among others, ATF6α induces the expression of chaperones and UPR mediators including *BiP* and *X-box binding protein 1* (*Xbp1*) contributing to proteostasis and augmentation of the regulatory output of the IRE1 arm, respectively (Table 1). Other known targets within the regulatory repertoire of ATF6 include ER degradation-enhancing α-mannosidase-like protein 1 (EDEM1) and protein disulphide isomerase-associated 6 (PDIA6), which promote degradation of misfolded proteins [10]. Furthermore, cogent evidence has been demonstrated for the role of ATF6α in cytoprotection and growth-modulation [11]. Interestingly, deletion of both ATF6α and ATF6β in mice results in embryonic lethality whereas deficiency in either of these ATF6 homologues fails to cause this effect [12]. To date, few studies have examined the interaction between ATF6α and ATF6β in developmental and pathophysiological milieux. Furthermore, little is known about the contribution of the ATF6β isoform to the development and maintenance of various tissue types. Thus, this review will mainly highlight findings relevant to the role of ATF6α in development and homeostasis. Nevertheless, recent studies have demonstrated that ATF6β contributes to chondrogenic and adipogenic processes (as outlined later in this review) [13, 14]. Notably, loss of ATF6α increases disease severity in a mouse model of metaphyseal chondrodysplasia type Schmid, whereas ablation of ATF6β attenuates pathology [13]. Given the apparent paradoxical effects of both ATF6 isoforms in modulating disease severity, and embryonic lethality resulting from deficiency in both isoforms, further studies are warranted to investigate mechanisms underlying reciprocal modulation of ATF6α and ATF6β activity. Such endeavours will advance our understanding of ATF6 and ER stress biology and also yield insights relating to the role of ATF6β in human health and disease.

Additional ER membrane-tethered bZIP family transcription factors, such as BBF2H7, CRE-binding protein H (CREB-H), Luman and OASIS (old astrocyte specifically induced substance; also known as CREB3L1), may also be subjected to S2P-catalysed regulated intra-membrane proteolysis (RIP) in response to ER stress conditions. However, the functions of these alternative ATF6 family members are ill-defined [15]. Nevertheless, a framework is emerging regarding the molecular dynamics and diversity of these bZIP transcription factors. Conceivably, the ostensible facility of ATF6 proteins in regulating distinct cellular programmes (including growth, survival and differentiation) has cultivated an interest in exploring the significance of these proteins in development and tissue homeostasis, both in physiological and pathophysiological contexts.

### The emerging role of ATF6 in development

#### Osteogenesis and chrondrogenesis

**Fig. 1:**
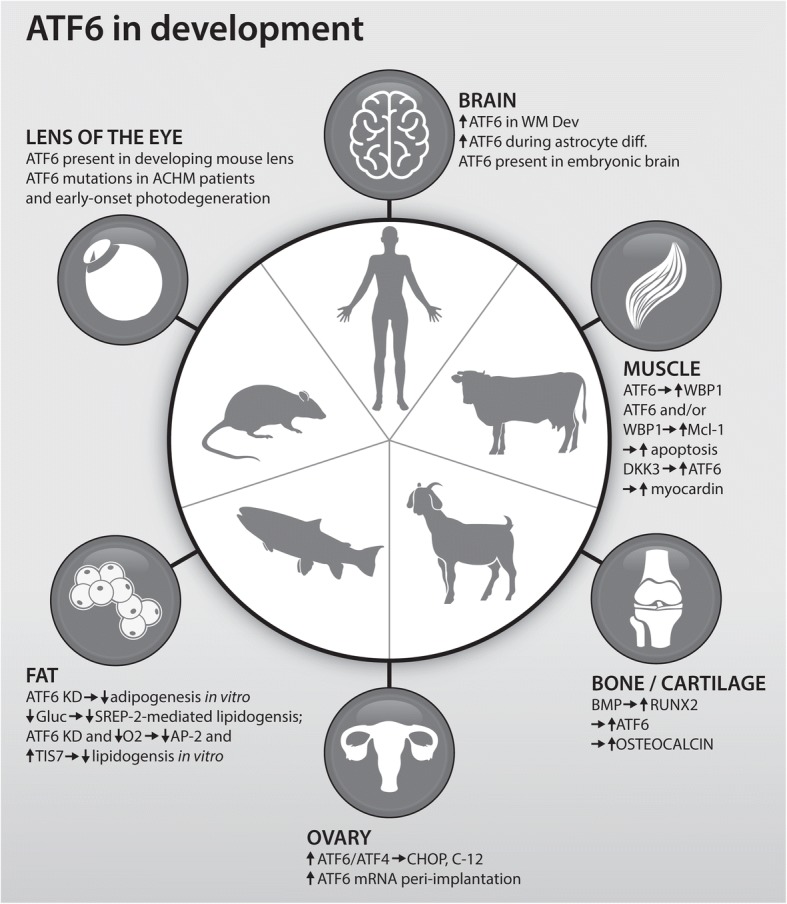
ATF6 in Development. Mutations in, molecules upstream or downstream from, ATF6, which are implicated in brain, muscle, bone, uterine and lens development, as well as adipogenesis

During vertebrate embryogenesis, skeletal structures initially are formed on a cartilaginous model. Thus, the molecular and cellular events regulating osteogenesis and chondrogenesis are tightly connected, both spatially and temporally [16]. ATF6 expression has been demonstrated in growth plate chondrocytes in vivo as well as during chondrocyte differentiation in pluripotent murine stem cell lines in vitro (C3H10T1/2 and ATDC5 cells) [17, 18]. Furthermore, overexpression of ATF6 enhances chondrocyte hypertrophy, mineralisation, and endochondral bone in foetal mouse metatarsals ex vivo [18]. Through adenoviral-mediated delivery of ATF6α siRNA (small interfering RNA) to C3H10T1/2 cells, Guo et al. [17] showed that ATF6 knockdown suppressed hypertrophic chondrocyte differentiation (Table 2, Fig. 1). Notably, bone morphogenetic proteins (BMPs), in particular BMP2, execute crucial chondrogenic and osteogenic functions. Interestingly, BMP2 stimulates osteoblast differentiation and mineralisation through Runt-related transcription factor 2 (Runx2)-induced ATF6 expression, which, in turn, promotes transcription of osteocalcin (the most abundant non-collagenous protein in bone) [19]. Additionally, Guo et al. [17] demonstrated that ATF6α augments Runx2-mediated chondrocyte hypertrophication in vitro and alters the crucial Indian hedgehog/parathyroid hormone-related peptide chondrogenesis pathway [17]. Recently, the ATF6β isoform was shown to promote murine growth plate chondrocyte proliferation [13]; further studies are merited in order to define the role of ATF6β in chondrogenic and other developmental processes.

Mineral trioxide aggregate (MTA) is frequently implemented as an endodontic cement. Treatment of MC3T3-E1 cells (pre-osteoblastic cell line) with MTA induces osteocalcin expression and mineralisation [20]. Following exposure of MC3T3-E1 cells to MTA, Maeda et al. [20] observed induction of *Atf6* mRNA expression and ATF6 activation. Furthermore, the authors provided evidence for a role for ATF6 in the induction of osteocalcin expression and MTA-induced mineralisation. Indeed, forced expression of ATF6, using a viral vector, significantly enhanced osteocalcin mRNA expression upon comparison to mock-transfected cells. Additionally, chromatin immunoprecipitation data revealed an increase in the binding of activated ATF6 to the osteocalcin promoter region following MTA treatment. Knockdown of ATF6 expression in MC3T3-E1 cells attenuated MTA-induced osteocalcin expression and osteoblastic mineralisation [20]. Together, these data demonstrate that MTA may induce in vitro osteoblastogenesis via an ATF6-osteocalcin axis. Moreover, overexpression of ATF6 in human dental pulp cells indicated that this transcription factor regulates odontoblastic differentiation and matrix mineralisation [21]. Mice deficient in another bZIP transcription factor, OASIS, exhibit impaired bone formation [22]. However, while conditional knockout of S1P in mice precipitates severe chondrodysplasia and abolishes endochondral ossification, the activity of ATF6 does not appear to be compromised in such genetic contexts [23]. Thus, an essential yet fragmentary portrayal of the importance of ATF6 and other bZIP transcription factors in the development of bone and cartilage is emerging, which may foster the development of novel regenerative and endodontic therapies.

#### Ocular embryology

The lens of the eye possesses two distinct cell types: epithelial cells in the anterior portion and fibre cells which are established continuously following differentiation of epithelial precursor cells at the lens equator. Indeed, fibre cell development is associated with pronounced membrane protein synthesis; thus, it was hypothesised that UPR mediators may be activated during lens development. The expression and cleavage of ATF6 was observed in nascent murine fibre cells, suggesting that this transcription factor is activated in the differentiation of fibre cells during embryogenesis (Table 2, Fig. 1) [24]. Interestingly, ATF6 mutations recently were implicated in the pathogenesis of the autosomal recessive cone dysfunction disorder achromatopsia (ACHM). ACHM is an early-onset, retinal dystrophy characterised by reduced visual acuity, pendular nystagmus, photophobia and colour blindness. Homozygosity mapping, linkage analyses and exome sequencing in a consanguineous Pakistani family with ACHM allowed for the identification of a single-base insertion *ATF6* variant which was predicted to result in a truncated, non-functional ATF6 product [25]. Furthermore, eight *ATF6* mutations were identified in ten families with ACHM, all of which caused an attenuation of ATF6 transcriptional activity. Patients displayed foveal hypoplasia and poorly formed or absent foveal pits, thereby distinguishing *ATF6*-associated ACHM from other forms of the disease in which foveal development typically is preserved [26]. Thus, ATF6 plays a crucial role in the development and functionality of the fovea and cone photoreceptors in humans (Table 2, Fig. 1). Additionally, biallelic loss-of-function *ATF6* mutations were identified in a two-year-old patient with early onset, photoreceptor degeneration. Specifically, this patient exhibited stop-gained (results in truncated protein) and splicing (results in abnormal mRNA transcript) variants on the paternal and maternal alleles, respectively, severely compromising ATF6 function. Moreover, this patient was too young to complete a colour vision test, however, observed foveal aberrations highly corresponded with those observed in ACHM patients [27]. Fortuitously, the peripheral cones may be present in *ATF6*-associated ACHM patients, potentially creating opportunity for gene therapy.

Recently, Kroeger et al. [28] demonstrated that treatment of human embryonic stem cells with a novel small molecule ATF6 activator (AA147) suppresses pluripotency, enhances stem cell differentiation and steers differentiating cells towards a mesodermal fate. Furthermore, induced pluripotent stem cells derived from patients carrying loss-of-function ATF6 mutations retain pluripotency and exhibit impaired mesodermal differentiation [28]. Together, these lines of evidence highlight a novel role for ATF6 in early stem cell development and mesodermal differentiation. This finding also aids in clarifying the pathophysiology underlying ocular disease as a consequence of impaired ATF6 function in humans. Indeed, deficient ATF6-mediated mesodermal differentiation may contribute to the precipitation of abnormal retinal vascular development and resultant visual disorders. At present, the molecular players involved in ATF6-mediated ocular development merit elucidation, both in physiological and pathophysiological states. Such mechanistic insights may propel the development of novel ophthalmological therapies.

#### Neuroembryogenesis

Presently, documentations of the role of bZIP family transcription factors in the development of the nervous system are lacking; nevertheless, exciting progress has been made. OASIS-deficient mice display fewer astrocytes in cerebral cortices during embryonic development, upon comparison to wild-type littermates. Furthermore, astrocyte differentiation is delayed in primary cultured neural precursor cells which lack OASIS [29]. The loss of OASIS-mediated induction of the transcription factor Gcm1 was proposed to result in protracted methylation of the *Gfap* promoter and impaired astrocyte differentiation. Interestingly, the activity of ATF6, as well as other UPR axes, was increased during astrocyte differentiation and such mild ER stress may permit OASIS induction and thus, promote astrocyte generation [29]. Upon comparison with adult tissues, the expression of a partially glycosylated form of ATF6 is elevated in the embryonic brain of mice [30]. Of note, while ATF6 activation occurred during differentiation of bone marrow stromal cells into neurons in vitro, ER stress appeared to be induced at a potency which was insufficient to elicit apoptosis [31]. ATF6 is also expressed at low levels in murine olfactory sensory neurons during development, exhibiting a 60-fold lower expression level than the transcription factor ATF5 in the main olfactory epithelium [32]. Strikingly, ATF6 is activated in oligodendrocytes during myelination in the developing rat cerebellum and nuclear-localised ATF6 peaks at postnatal day 10, coincident with the onset of myelination (Table 2, Fig. 1) [33]. Therefore, ATF6 appears to play a role in neuroembryogenesis and myelinogenesis, in that its expression peaks during periods of embryonic and early postnatal development. While further evidence is warranted, preliminary evidence suggests that modulation of ATF6-mediated UPR signals may represent a novel strategy encompassing neuroreparative and/or neuroprotective elements.

#### Embryology of the muscular system

In developing murine muscle tissue, ATF6 induces apoptosis through the activation of caspase-12 [34]. Moreover, ATF6, possibly via the ww-domain-binding protein 1 (WBP1), is implicated in the reduction of cellular levels of the anti-apoptotic protein myeloid cell leukaemia sequence 1 (Mcl-1) in apoptotic myoblasts; however, quantitative polymerase chain reaction analyses indicate that ATF6 does not downregulate Mcl-1 expression at the level of transcription (Table 2, Fig. 1). Therefore, ATF6 may promote post-translational degradation or modification of Mcl-1 in apoptotic cells during embryonic muscle development [35]. Furthermore, dickkopf homolog 3 (DKK3)-induced expression of ATF6 promoted the differentiation of mouse embryonic stem cells into smooth muscle cells via ATF6-mediated upregulation of the smooth muscle cell transcription factor, myocardin (Table 2, Fig. 1) [36]. Thus, ATF6 signalling may play a role in developmental apoptosis and differentiation programmes during embryonic muscle development in mice. At present, the extent to which ATF6 participates in the embryonic development of the human muscular system is unknown.

#### Development of female reproductive structures

In relation to reproductive physiology, ATF6α may execute crucial apoptotic functions during the late-luteal phase in rat corpus luteum regression through the ATF6α-CHOP and caspase 12 pathways, although it remains possible that CHOP induction is mediated by other transcription factors, such as XBP1 or ATF4 [37]. Additionally, ATF6 and ATF4-induced CHOP expression reported in bovine ovarian tissue may also affect granulosa cell differentiation in corpus luteum biogenesis as well as apoptosis during corpus luteum regression [38]. Notably, ATF6 has also been implicated in apoptosis of ovarian goat granulosa cells during follicular atresia [39]. Therefore, this UPR transducer may serve as a crucial regulator in the development of ovarian structures, in particular during follicular physiological processes (Table 2, Fig. 1). Moreover, documentations of increased ATF6α expression in murine uterine tissue suggested that ATF6 may also regulate embryo implantation and decidualisation (the process of forming decidua: the uterine lining during pregnancy and maternal part of placenta) [40].

#### Adipogenesis and lipogenesis

Knockdown of ATF6α impaired the differentiation of an adipogenic cell line into mature adipocytes in vitro, as well as associated lipogenesis, highlighting the potential importance of ATF6 in adipogenic and lipogenic processes (Table 2, Fig. 1) [41]. On the other hand, ATF6 may suppress sterol regulatory element-binding protein (SREBP)-2-mediated lipogenesis under conditions of glucose deprivation (SREBP-2 is also a S1P/S2P substrate) [42]. Moreover, the expression of ATF6 proteins is upregulated during salmon (ATF6) and rainbow trout (ATF6β) adipogenesis [14, 43]. Notably, these findings in salmon and rainbow trout demonstrate that both isoforms of ATF6 may regulate tissue development (in particular, development of adipose tissue); indeed, further studies are merited in order to clarify the precise contribution of each isoform to developmental processes. Future research efforts should also focus on determining whether ATF6α and ATF6β have synergistic or antagonistic effects with respect to one another in the context of developmental operations, such as during adipo−/lipogenesis. Additionally, few studies have examined the role of ATF6β isoform in tissue development and homeostasis necessitating investigation of this ER stress protein in adipogenic, lipogenic, and other developmental, processes in higher organisms. Interestingly, knockdown of ATF6 significantly attenuated hypoxic stress-induced reductions in adipogenic gene expression, specifically *adipocyte protein 2* (AP-2), in pre-adipocytes. Indeed, ATF6 knockdown also ameliorated hypoxia-induced upregulation of the transcriptional repressor tetradecanoyl phorbol acetate induced sequence 7 (TIS7) [44]. Therefore, during hypoxic stress conditions, ATF6 may upregulate TIS7 expression prompting an inhibition of adipogenesis in pre-adipocytes in vitro (Table 2, Fig. 1); however, the relevance of ATF6-mediated adipogenesis inhibition in human cells exposed to hypoxic environments merits elucidation. Hence, ATF6 signalling may positively regulate adipogenesis and lipogenesis; however, ATF6 proteins may also counteract adipogenic and lipogenic systems in settings of cellular stress in order to ensure energy conservation.

### ATF6 signalling in tissue homeostasis and disease

Seminal studies revealed that ATF6α signalling promotes resistance to acute and chronic stress in mouse embryonic fibroblasts, suggesting that ATF6 fosters tissue homeostasis and stress remediation in vertebrate systems [45]. Recently, an expanding body of evidence has implicated the ATF6 pathway in the maintenance of tissue homeostasis, such that deregulated ATF6 signalling in adult organisms may precipitate devastating clinical consequences (see Table 3). For instance, ATF6α activity fosters hepatocarcinogenesis [46]. Furthermore, ATF6α-mediated activation of Rheb and mTOR signalling promotes survival of dormant human squamous carcinoma cells (D-HEp3 cells) and their adaptation to chemotherapy and the in vivo microenvironment [47]. Thus, disruption of the ATF6α-Rheb-mTOR pathway may permit the eradication of residual disease. In the heart, ATF6α governs cardiomyocyte survival responses to growth stimuli and may protect against the development of pathological cardiac hypertrophy and heart failure [48]. Additionally, cardiac tissue from ATF6-knockout mice showed increased damage and decreased function following myocardial ischemia/reperfusion injury, upon comparison to wild-type littermate tissue [49]. Therefore, ATF6α may exert protective effects in the adult heart. Calcium-independent phospholipase A2γ (iPLA2γ) is cytoprotective against glomerular epithelial cell (GEC) injury which provokes the development of proteinuria in glomerular disease states. Recently, iPLA2γ-mediated GEC cytoprotection was shown to be dependent on ATF6 signalling which may limit GEC injury in proteinuric glomerular diseases [50]. Similarly, ATF6 executes cytoprotective functions in nervous tissue. Indeed, conditional and tamoxifen-induced forced activation of ATF6 in forebrain neurons reduced infarct volume and improved functional recovery in a mouse model of stroke [51]. Moreover, the ATF6 branch of the UPR is associated with hippocampal and striatal neuroprotection in models of Huntington’s disease and neurotoxicity, respectively [52, 53]. In addition, small molecule ATF6 activation corrects proteostatic imbalances and attenuates inappropriate secretion and aggregation of, for instance, amyloidogenic proteins in disorders such as systemic amyloidosis which affects various organ systems [54]. Fascinatingly, ATF6α both promotes and prevents the development of diabetes in mice as diet-induced obese ATF6α-knockout mice exhibit glucose intolerance due to pancreatic β-cell failure but are partially resistant to diet-induced insulin resistance [55]. In addition, ATF6 was detected at increased levels in the islet cells of diabetic Otsuka Long Evans Tokushima Fatty rats, when compared to non-diabetic controls [56]. In a zebrafish model of fatty liver disease, ATF6 prevents hepatic steatosis following tunicamycin-induced acute ER stress whereas ATF6 potentiates steatosis due to chronic ER stress [57].

In summary, ATF6α serves as an important homeostatic regulator operating in cell- and tissue-specific contexts, and aberrant ATF6 signalling may promote pathogenesis of diverse disease states, including cancer.

## Conclusions and future directions

Classically, ATF6 has been linked primarily to cellular processes aimed at relieving the build-up of misfolded proteins that occurs during pathological ER stress. However, an increasing body of literature is pointing to a critical role in normal development and tissue homeostasis. Molecules reported to be induced by ATF6 or those that exploit ATF6 are varied, depending on the tissue context. For example, the RunX2-ATF6-osteocalcin axis is implicated in bone and cartilage development. WP1, MCl-1 and DKK3 have been identified in ATF6-mediated muscle development. SREBP2, AP-2 and TIS7 levels are altered following manipulation of ATF6 expression during adipo/lipogenesis, and reproductive or brain developmental processes cause upregulation of canonical markers of ER stress, such as CHOP, caspase-12, and ER chaperones. Despite these exciting results, it is intriguing to note that descriptions of the contribution of ATF6 in the development of certain anatomical structures such as gastrointestinal and lung tissues are entirely lacking.

The critical importance of ATF6-regulated processes to human health is exemplified by the occurrence of achromatopsia in individuals harbouring ATF6 mutations. Furthermore, the cytoprotective properties of ATF6 have been demonstrated in the context of human disorders ranging from cardiac and brain ischemia, to glomerular disease. Some caution is recommended, however, as ATF6-mediated effects are controversial in the context of diabetes and may promote the survival of liver and squamous cell carcinomas. In conclusion, it is clear that only some of the pieces of the ATF6 puzzle are in place and that significant gaps in knowledge remain. The translational potential of molecular manipulation of ATF6 expression using, for example, small molecular weight compounds [54] or genetic manipulation has yet to be realised and must be explored by further investigations.

## Abbreviations

ACHM: Achromatopsia
ATF6: Activating transcription factor 6
BiP: Binding immunoglobulin protein
BMP: Bone morphogenetic protein
bZIP: Basic leucine zipper
CHOP: C/EBP homologous protein
CREB: CAMP responsive element-binding protein
EDEM1: ER degradation enhancing alpha-mannosidase like protein 1
eIF2α: Eukaryotic initiation factor 2α
ER: Endoplasmic reticulum
GEC: Glomerular epithelial cell
iPLA2γ: Calcium-independent phospholipase A2γ
IRE1: Inositol-requiring enzyme 1
MCL-1: Myeloid cell leukaemia sequence 1
MTA: Mineral trioxide aggregate
OASIS: Old astrocyte specifically induced substance
PDIA6: Protein disulphide isomerase-associated 6
PERK: Protein kinase RNA-like endoplasmic reticulum kinase
RIP: Regulated intramembrane proteolysis
RunX2: Runt-related transcription factor 2
S1P/S2P: Site-1/2 protease
TIS7: Tetradecanoyl phorbol acetate induced sequence 7
UPR: Unfolded protein response
XBP1: X-box binding protein 1
